# Evaluation of dry textile electrodes for long-term electrocardiographic monitoring

**DOI:** 10.1186/s12938-021-00905-4

**Published:** 2021-07-12

**Authors:** Milad Alizadeh-Meghrazi, Binbin Ying, Alessandra Schlums, Emily Lam, Ladan Eskandarian, Farhana Abbas, Gurjant Sidhu, Amin Mahnam, Bastien Moineau, Milos R. Popovic

**Affiliations:** 1 KITE Research Institute, Toronto Rehabilitation Institute - University Health Network (UHN), Toronto, ON Canada; 2 The Institute for Biomedical Engineering, University of Toronto, Toronto, ON Canada; 3 Department of Mechanical Engineering, McGill University, Montreal, QC Canada; 4 Department of Mechanical and Mechatronics Engineering, University of Waterloo, Waterloo, ON Canada; 5 Department of Materials Science and Engineering, University of Toronto, Toronto, ON Canada; 6 Department of Chemistry, University of Toronto, Toronto, ON Canada; 7 Department of Nanotechnology Engineering, University of Waterloo, Waterloo, ON Canada; 8 Myant Inc., Toronto, ON Canada

**Keywords:** Dry textile electrodes, Electrophysiological monitoring, Electrocardiography (ECG), Textile computing, Long-term biosignal monitoring, Remote healthcare, Printed electronics, Carbon-contained yarn, Silver-plated yarn

## Abstract

**Background:**

Continuous long-term electrocardiography monitoring has been increasingly recognized for early diagnosis and management of different types of cardiovascular diseases. To find an alternative to Ag/AgCl gel electrodes that are improper for this application scenario, many efforts have been undertaken to develop novel flexible dry textile electrodes integrated into the everyday garments. With significant progresses made to address the potential issues (e.g., low signal-to-noise ratio, high skin–electrode impedance, motion artifact, and low durability), the lack of standard evaluation procedure hinders the further development of dry electrodes (mainly the design and optimization).

**Results:**

A standard testing procedure and framework for skin–electrode impedance measurement is demonstrated for the development of novel dry textile electrodes. Different representative electrode materials have been screen-printed on textile substrates. To verify the performance of dry textile electrodes, impedance measurements are conducted on an agar skin model using a universal setup with consistent frequency and pressure. In addition, they are demonstrated for ECG signals acquisition, in comparison to those obtained using conventional gel electrodes.

**Conclusions:**

Dry textile electrodes demonstrated similar impedance when in raised or flat structures. The tested pressure variations had an insignificant impact on electrode impedance. Looking at the effect of impedance on ECG signals, a noticeable effect on ECG signal performance metrics was not observed. Therefore, it is suggested that impedance alone is possibly not the primary indicator of signal quality. As well, the developed methods can also serve as useful guidelines for future textile dry-electrode design and testing for practical ECG monitoring applications.

**Supplementary Information:**

The online version contains supplementary material available at 10.1186/s12938-021-00905-4.

## Background

Biopotentials or electrophysiological signals are widely utilized in health monitoring and diagnostics. For example, through electrocardiography (ECG), cardiac activity is recorded and information regarding potential cardiovascular diseases (CVD) (e.g., heart rhythm abnormalities including atrial fibrillation) can be obtained [1]. To record ECG signals, biopotential electrodes are used, which are classified as invasive, that puncture the skin, or non-invasive, that do not puncture skin. With invasive electrodes, the extracellular and intracellular fluids serve as an electrolytic medium [2] for improved signal acquisition. Due to the nature of these electrodes, requiring the puncturing of the skin, they are not ideal for long-term continuous monitoring. As well, they present a further limitation with implementation, requiring the supervision of a trained professional. With non-invasive electrodes, an electrolytic gel can be used, or they can be used without electrolytic gel, considered as dry contact electrodes. Currently in standard clinical settings, Ag/AgCl gel electrodes are mainly used for recording ECG signals. These types of electrodes use a conductive gel that acts as an electrolyte between the electrode and the epidermis layer of skin, and reduces the contact impedance between them [3]. As well they have an adhesive material around the perimeter of the electrode, that helps with adherence to skin, reducing movement of the electrode, improving signal quality during motion. However, issues such as skin irritation [4] over time, and decay of signal quality due to gel dehydration [5, 6], hinder them from continuous and long-term wearable monitoring usage. Alternatives to the hydrogel adhered electrodes can be realized through the manual application of electrolytic hydrogel, or electrolytic pastes. These solutions leave a feasibility gap with their application and durability, limiting their consideration for continuous monitoring. As the importance of continuous long-term ECG monitoring is increasingly recognized for early diagnosis and management of different types of CVD, significant effort has been devoted to the development of skin-friendly dry electrodes for wearable biopotential measurements [7–12].

**Fig. 1:**
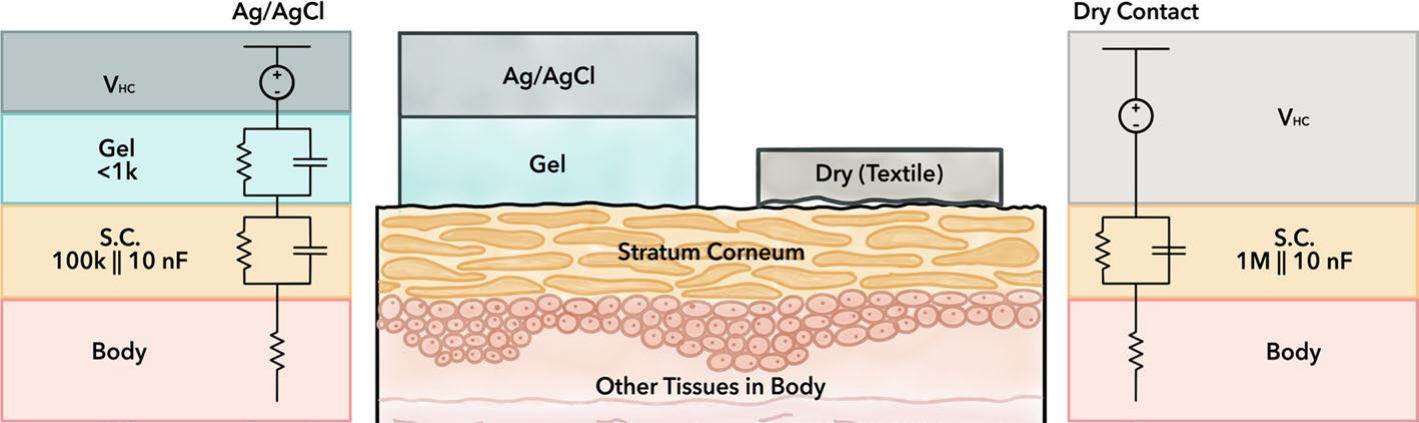
Electrode–skin interface with **a** gel electrode vs. **b** dry contact electrode. Demonstrating differences in stratum corneum impedance electrical model when exposed to the moisture of electrolyte gel [31]

Flexible textile dry electrodes have been recently the focus of many researches, with the aim to convert biopotential monitoring to a nonintrusive process achieved with the comfort of the everyday garments that we are already using [9, 10]. ECG electrodes are mainly fabricated using different conductive materials, such as metals [13–16], intrinsically conductive polymers (ICPs) [8, 17], and carbon-based materials [18–23]. These conductive materials and yarns can be integrated into fabrics through conventional manufacturing methods (e.g., knitting, weaving, embroidery) [24–26], or can be applied onto textiles through various techniques (e.g., stenciling, screen printing, and sputtering) [27]. Many efforts have been undertaken to optimize those novel dry electrodes, addressing issues such as weak signal pick-up, low signal-to-noise ratio, high skin–electrode impedance, motion artifact, as well as biocompatibility, and durability [10]. Among them, impedance reduction is one of the most intensively studied research directions [7, 28, 29]. In dry contact electrodes, high skin–electrode contact impedance is observed. This is primarily due to the lack of electrolytic gel or skin preparations at the electrode application site, such as removing hair or abrading with sand paper and cleaning with alcohol wipes (lightly removing the stratum corneum). The electrical circuit model for the skin–electrode interface can be represented by resistive and capacitive components (Fig. 1) [2]. Both electrolytic gel and skin preparation are meant to reduce the skin–electrode contact impedance, which have demonstrated to improve signal acquisition quality, although it is unknown if low electrode impedance alone can guarantee high-quality biopotential recording. For example, organic conductive poly(3,4-ethylenedioxythiophene)-poly(styrenesulfonate) and Ionic Liquid PEDOT:PSS + IL gel were directly printed on knitted textiles, showing a low contact impedance, and was utilized for long-term ECG recording [8]. With a variety of dry textile electrodes proposed by different research labs to date (Additional file 1: Table S1), it is very difficult to understand which methods and materials lead to high-performance textile electrodes that can potentially be used in practical “out of the lab” situations. This is mainly because previous studies have usually utilized different testing platforms and parameters (e.g., different textile substrates, fabrication methods, conductive coating materials, applied pressures, frequencies, skin characteristics, and ambient conditions) to evaluate and compare electrodes. Thus, we need a universal/standard evaluation protocol for electrode design and selection efficiently in the practical application scenarios [30].

In this paper, we aim to compare the electrical performance of various existing dry textile electrodes using a universal setup with consistent frequency, pressure, and skin model. Different representative electrode materials [e.g., carbon, IL, polydimethylsiloxane (PDMS)/(PEDOT:PSS), PDMS/PEDOT:PSS/IL, PDMS/PEDOT:PSS/carbon nanotube (CNT)] reported previously have been deposited onto a flat/raised silver or carbon textile substrates by screen printing. Screen-printing was utilized for electrode fabrication because it is a simple and versatile technique well-suited and commonly used for mass production in the textile industry. To verify the performance of dry textile electrodes, impedance measurements were conducted on an agar skin model, while ECG signals were also acquired, comparing the signal fidelity to those obtained using conventional gel electrodes. These results can serve as useful guidelines for future textile-based dry-electrode design and practical ECG monitoring applications.

## Results

### Dry textile electrode structures and their coated conductive materials

In this study, several different materials were used to create electrodes using screen printing on flat and raised, silver and carbon knitted electrodes. Then the impedance of these electrodes to a skin model was measured, and ECG was recorded by these electrodes, to compare their performances to gel electrodes as well as bare silver and carbon electrodes. Silver-plated nylon and carbon-contained nylon conductive yarns (Myant Inc., Canada) were knitted into four different structures, namely, flat textile electrodes made of silver yarn (*FS* Sample code), raised 3D textile electrodes made of silver yarn (*RS* Sample code), flat textile electrodes made of carbon yarn (*FC* Sample code) and raised 3D textile electrodes made of carbon yarn (*RC* Sample code) using a flatbed knitting machine (Stoll CMS ADF 32W E7.2, Reutlingen, Germany).

**Fig. 2:**
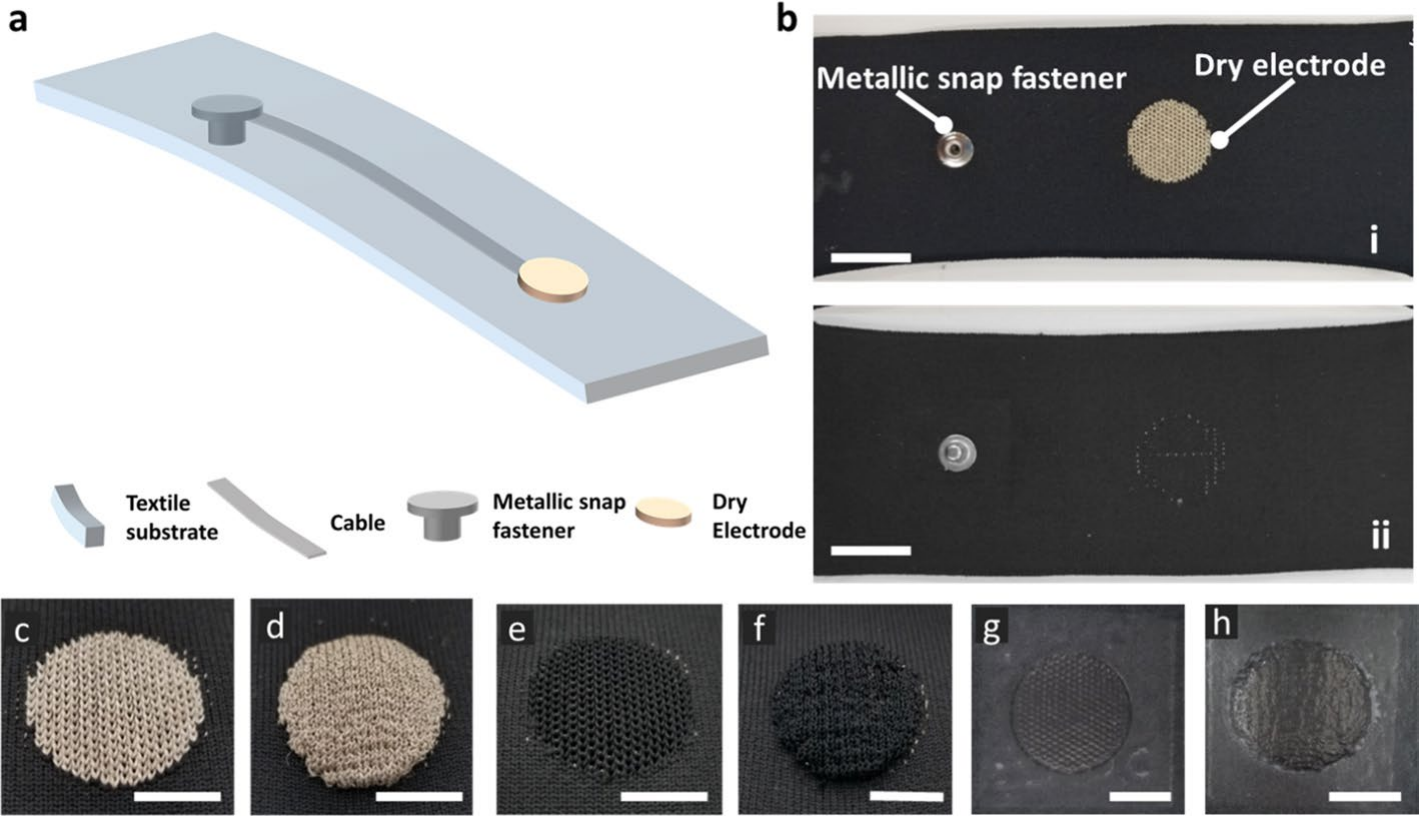
Dry textile electrodes. **a** Schematic of a dry textile electrode. **b-i** The front and **b-ii** back of a sample dry textile electrode. Scale bar is 2 cm. **c** Flat textile electrode made of silver yarn (FS). **d** Raised 3D textile electrode made of silver yarn (RS). **e** Flat textile electrode made of carbon yarn (FC). **f** Raised 3D textile electrode made of carbon yarn (RC). **g** Flat textile electrode with screen-printed coating. **h** Raised 3D textile electrode with screen-printed coating. Scale bar is 1 cm

Each textile electrode has a circular structure with a diameter of 2 cm, matching the active area of the hydrogel electrodes. Figure 2 shows a schematic of knitted textile swatches and the physical appearance of the flat/raised textile electrodes. A knitted silver-plated nylon trace/cable was used as a connection line between the knitted flat/raised electrode area and an electrode snap located on the back of the sample (Fig. 2a, b). Figure 2c–f shows images of the flat and raised textile electrodes made of conductive silver and carbon yarns, respectively.

Thereafter, different conductive pastes (20 types) were screen-printed on these 4 types of textile electrodes (namely FS, RS, FC, and RC, shown in Fig. 2c–f). Details of conductive pastes and formulations are described in “Methods” section.

**Fig. 3:**
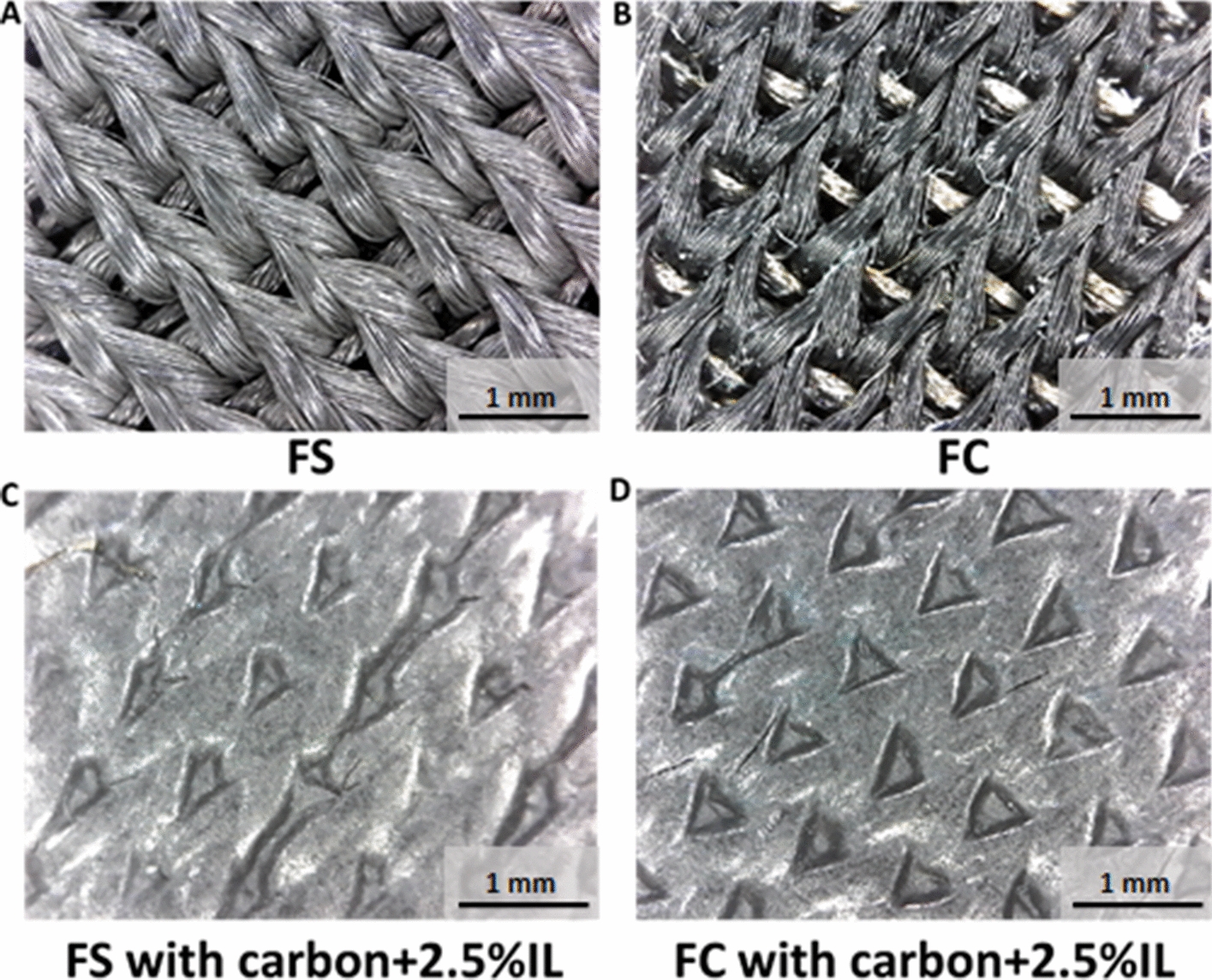
Microscopic images of dry textile electrodes. **a** FS structure. **b** FC structure. **c** FS structure with carbon + 2.5%IL coating. **d** FC structure with carbon + 2.5%IL coating

**Fig. 4:**
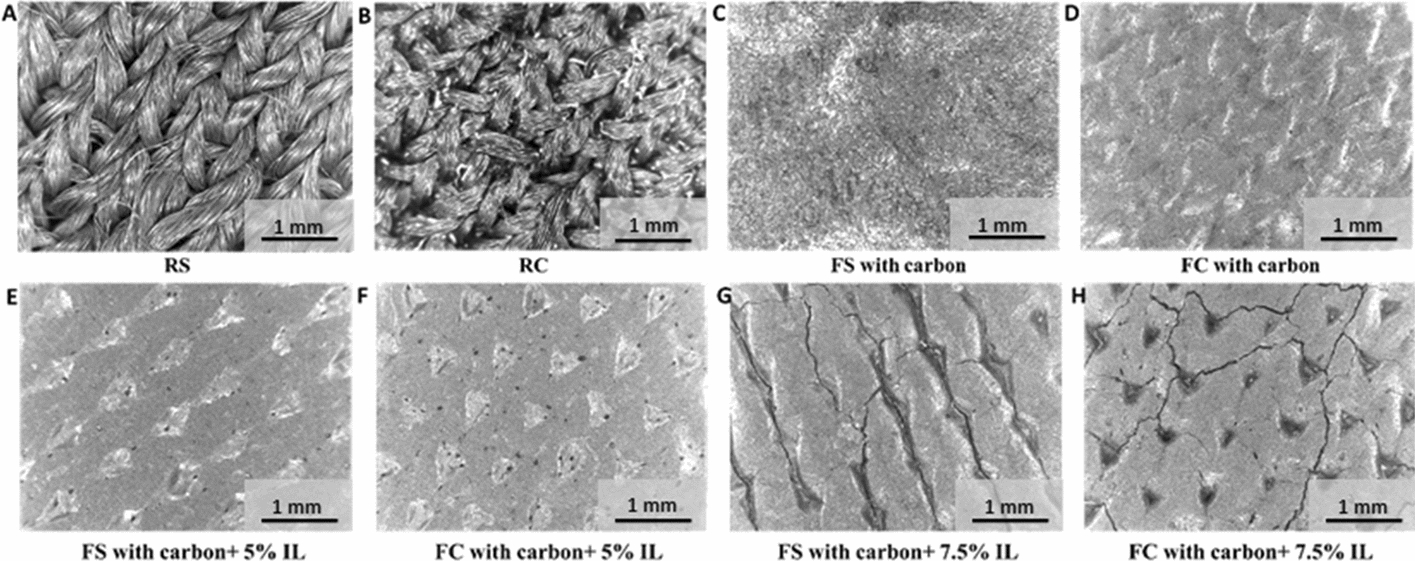
Microscopic images of textile electrodes. **a** RS structure. **b** RC structure. **c** FS structure with carbon coating. **d** FC structure with carbon coating. **e** FS structure with carbon + 5%IL coating. **f** FC structure with carbon + 5%IL coating. **g** FS structure with carbon + 7.5%IL coating. **h** FC structure with carbon + 7.5%IL coating

The performance of dry textile electrodes for biopotential monitoring is determined by a variety of factors (Table 1 and Additional file 1: Table S2), e.g., textile electrode substrates and structures, conductive coating materials for screen printing, applied pressures, and frequencies. In this section, we first explored the effect of electrode structures and coating conductive materials on the fabrication of dry textile electrodes. The dry textile electrodes were manufactured as flat or raised 3D structures (Fig. 2c–f). The flat structure allows for uniform pressure distribution, in line with the base fabric construction. Conversely, the raised 3D structures are protrusions from the base fabric construction leading to higher contact pressure, which could result in improved signal acquisition properties [11]. The microscopic images of FS (Fig. 2c) and FC (Fig. 2e) are shown in Fig. 3a, b. The microscopic images of RS (Fig. 2d) and RC (Fig. 2f) are shown in Fig. 4a, b, respectively. We can find a similar microscopic morphology between flat and raised 3D textile electrodes made of silver yarn (Fig. 3a, b), but a significant morphology difference between flat and raised 3D textile electrodes made of carbon yarn (Fig. 4a, b). Thereafter, we screen-printed conductive materials (listed in Table 1 and Additional file 1: Table S2) on these four types of textile substrates. Carbon-contained conductive coating materials (with/without IL) showed good printability on flat structures without obvious cracks formation (Figs. 3c, d, 4c–f). However, carbon-contained conductive coating materials with high concentrations of IL (e.g., 7.5%) showed cracks after screen printing on the flat textile substrates (Fig. 4g, h) and failed on the raised 3D textile substrates, which was excluded from implementation. The PEDOT:PSS + PDMS-contained conductive coating materials with/without CNTs or with a suitable concentration of IL (e.g., ≤ 5%) only showed printability without mechanical cracking on the flat textile substrates made of silver yarn. In contrast, the other combinations of PEDOT:PSS + PDMS-contained conductive coating materials listed in Additional file 1: Table S2, all failed when printed on the four textile substrates. For example, pristine PEDOT:PSS and PEDOT:PSS + IL could not be printed due to the observed mechanical cracks. This poor printability is due to the lack of flexibility and stretchability of PEDOT:PSS [32], which can be strengthened by applying biocompatible additives such as PDMS. In this scenario, we observed that PEDOT:PSS + PDMS-contained conductive coating materials without or with proper concentration of IL (e.g., ≤ 5%) could be printed on the flat textile substrates made of silver yarn. With further increase of IL concentration (e.g., 7.5%), this coating was observed not printable on the flat textile substrates made of silver yarn.

**Table 1:** Coating formulations successfully screen-printed onto dry textile electrodes

Coating material composition	Layers	Heat curing	Pass/fail
Carbon	3	140 °C, 2 min	Pass on FS, RS, FC, and RC^a^
Carbon + 2.5% IL^b^	3	140 °C, 2 min	Pass on FS, RS, FC, and RC
Carbon + 5% IL	3	140 °C, 2 min	Pass on FS, RS, FC, and RC
Carbon + 7.5% IL	3	140 °C, 2 min	Pass on FS and FC Fail on RS and RC
PEDOT:PSS + 12.5% PDMS	2	150 °C, 10 min	Pass on FS Fail on RS, FC, and RC
PEDOT:PSS + 6.25% PDMS	2	150 °C, 10 min	Pass on FS Fail on RS, FC, and RC
(PEDOT:PSS + 6.25% PDMS) + 2.5% IL	2	150 °C, 10 min	Pass on FS Fail on RS, FC, and RC
(PEDOT:PSS + 6.25% PDMS) + 5% IL	2	150 °C, 10 min	Pass on FS Fail on RS, FC, and RC
PEDOT:PSS + 12.5% (PDMS + 1% CNT)^c^	2	150 °C, 10 min	Pass on FS Fail on RS, FC, and RC
PEDOT:PSS + 6.25% (PDMS + 1% CNT)	2	150 °C, 10 min	Pass on FS Fail on RS, FC, and RC

^a^*FS* flat textile electrodes made of silver yarn, *RS* raised 3D textile electrodes made of silver yarn, *FC* flat textile electrodes made of carbon yarn, *RC* raised 3D textile electrodes made of carbon yarn

^b^Carbon-based coatings were mixed with 2.5% IL

^c^PEDOT:PSS-based coatings were mixed with 6.25% PDMS and 1% CNT

### Impedance of dry textile electrodes

Electrode impedance has been considered as one of the main performance metrics in evaluating electrodes for recording high-quality biosignals [7, 28, 29]. We evaluated the impedance performance of dry textile electrodes with and without conductive materials (listed in Table 1) screen-printed on them. An agar skin model was utilized to mimic human skin for consistency in the testing of the electrodes and the avoidance of intra- and inter-subject skin impedance variations [33].

#### Effect of frequency and coating materials

**Fig. 5:**
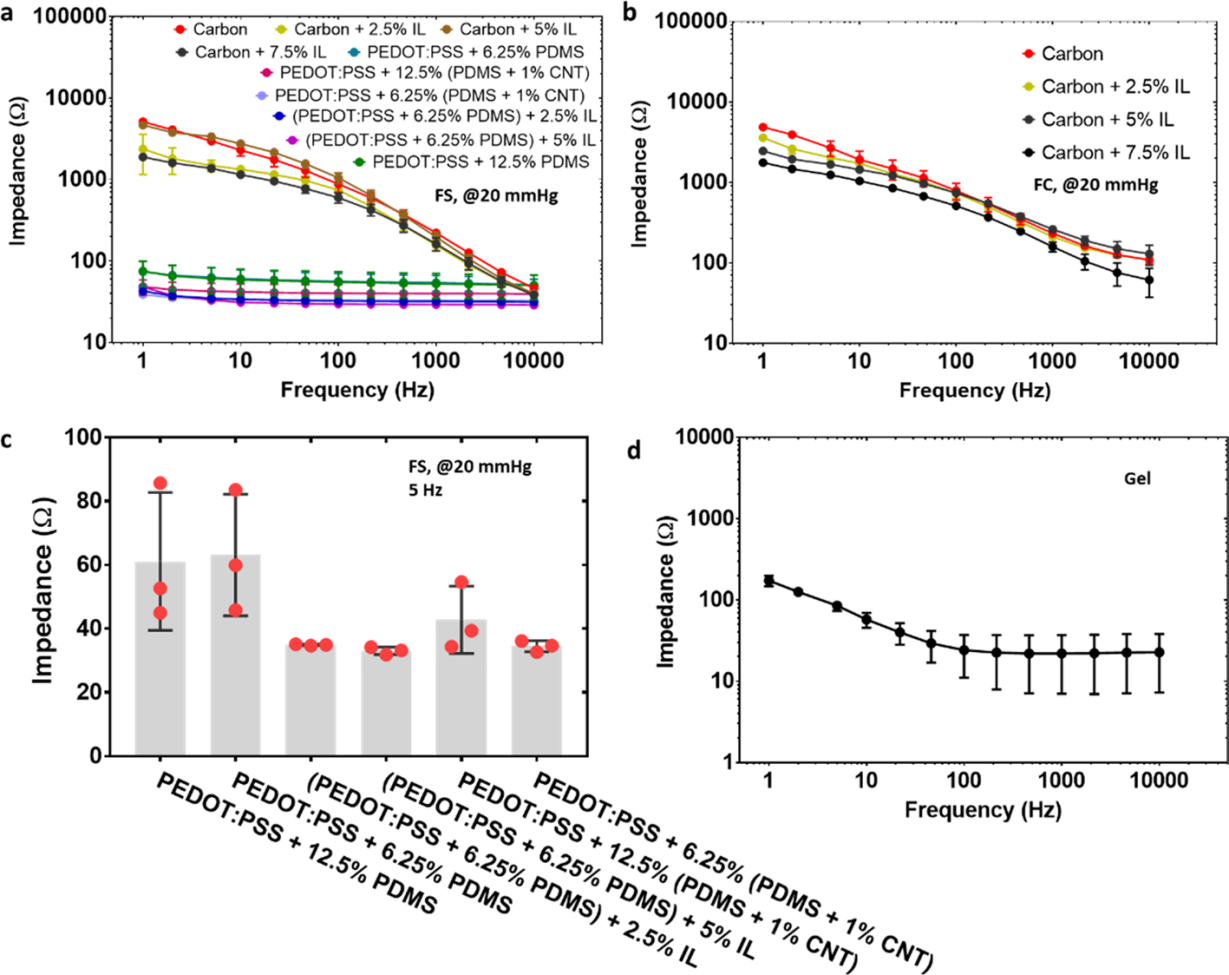
The agar–electrode impedance of textile electrodes. **a** FS structure with various conductive coating materials in the frequency range of 1–10,000 Hz. **b** FC structure with various conductive coating materials in the frequency range of 1–10,000 Hz. **c** FS structure with PEDOT:PSS-contained coating in the frequency of 5 Hz. The pressure was 20 mmHg (~ 2.66 kPa). **d** Wet gel electrode in the frequency range of 1–10,000 Hz

The agar–electrode impedance of different dry textile electrodes at a widely accepted frequency range (e.g., 1 Hz ~ 10 kHz [7, 9]) is shown in the bode plots (Fig. 5a, b and Additional file 1: Figure S3). All the tests were conducted under 20 mmHg (~ 2.66 kPa) (a typical pressure for wearable ECG measurement [34, 35]). The contact impedance decreased with the increase of frequency, which follows the typical trend of the electrical impedance measured on skin [36].

We then compared the agar–electrode impedance of dry textile electrodes with different conductive coating materials. Flat textile electrodes made of silver yarn were selected for comparison purposes since all conductive materials listed in Table 1 could be successfully printed on this type of textile substrate without cracks formation. As shown in Fig. 5a, the flat textile electrodes made of silver yarn with carbon-contained coating presented a much higher impedance than those with PEDOT:PSS-contained coating, showing a similar trend as previously reported [7]. In addition, the impedance decreasing rate highly depended on the coating conductive materials. Dry textile electrodes with carbon-contained coatings showed a much larger impedance change than that with PEDOT:PSS-contained coatings (Fig. 5a). Dry textile electrodes with PEDOT:PSS-contained coatings showed a comparable impedance level with that of standard gel electrodes (Fig. 5a, d). This could be explained by two facts: (a) carbon has a much higher sheet resistance than PEDOT:PSS [7]; (b) PEDOT:PSS can provide enhanced ionic-to-electric coupling effect, leading to a better impedance match with human skin [29]. In addition, we found that the impedance of dry textile electrodes with PEDOT:PSS-contained coatings can be further reduced through the adding of CNT or ionic liquid (Fig. 5c). The CNT-induced enhancement can be because CNTs suppress the phase separation of PEDOT:PSS. Namely, the nanotubes establish electrical interconnections between the separate PEDOT:PSS (conductive phase) islands being dispersed in the insulating PSS-phase, thereby enhancing the electrical conductivity [37, 38]. On the other hand, ILs as a secondary dopant can induce the formation of a nano-crystallized fibrillar structure of PEDOT chains with extended planar confirmations and reduced π–π interchain distances [39, 40]. There is no impedance difference among PEDOT:PSS-contained coating with various concentrations of PDMS containing CNTs or IL (Fig. 5c).

#### Effect of different conductive substrates (silver or carbon) and knitted structures (flat or raised 3D)

**Fig. 6:**
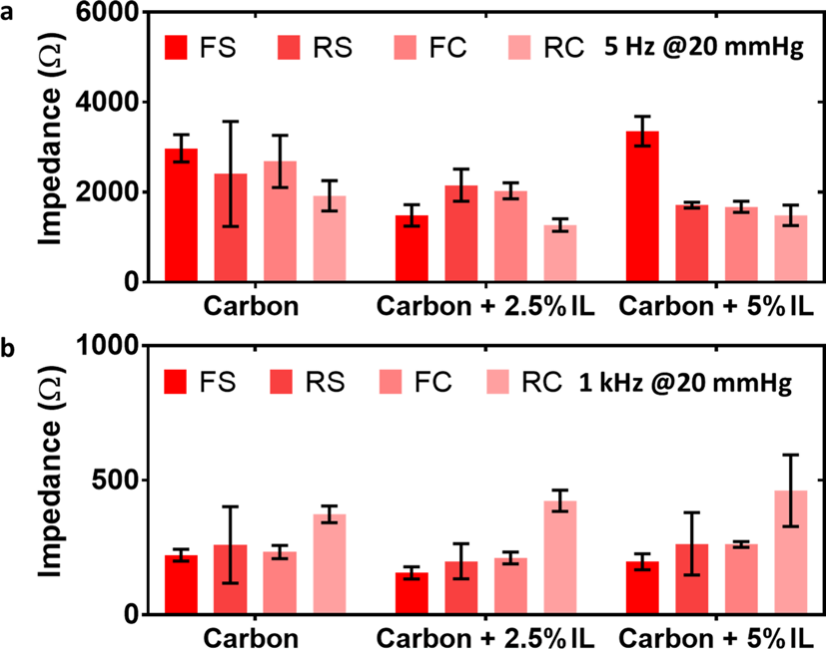
The agar–electrode impedance of dry textile electrodes. Different conductive substrates (silver and carbon) and knitted structures (flat and raised 3D) at low frequency (**a** 5 Hz) and high frequency (**b** 1 kHz)

In this study, we selected the dry textile electrodes with carbon-contained coatings for investigation because PEDOT:PSS + PDMS-contained coatings cracked on all dry textile substrates (RS, FC, and RC) except the FS structure. These coatings can only be printed on FS structure. In contrast, carbon-contained coatings could be printed on both flat and raised 3D structures without cracks formation due to the strength of covalent bonds between carbon atoms. All tests were conducted at 5 Hz because the ECG spectrum has been reported to present optimal signal amplitudes at the frequency of 4–5 Hz [41]. All carbon-contained coatings showed a similar impedance level on those four types of electrode structures; only the FS structure with (carbon + 5%IL) coating showed relatively high value of impedance (Fig. 6a) but still within the acceptable impedance range (e.g., ≤ 10 kΩ) of dry electrodes [7].

Dry textile electrodes have also shown a huge potential for epidermal electroencephalography (EEG) and electromyography (EMG) recording, in which tests were clinically conducted at higher sampling frequency ranges (e.g., 1 kHz) [36]. Therefore, we investigated the impedances of dry textile electrodes at the frequency of 1 kHz as well. As shown in Fig. 6b, all carbon-contained coatings showed similar impedance levels on FS, RS, and FC structures. Only carbon-contained coatings on the RC structure showed a relatively higher impedance (Fig. 6b).

#### Effect of pressure

**Fig. 7:**
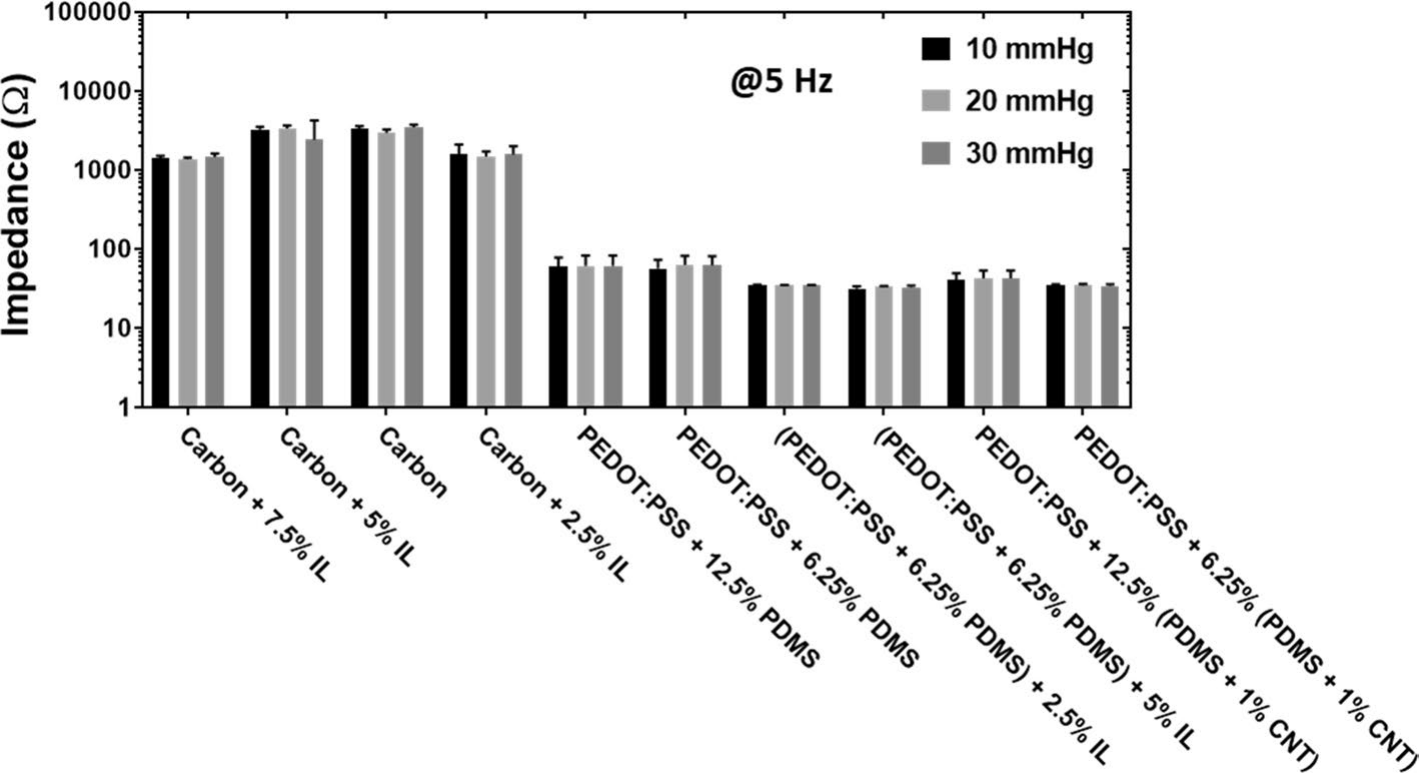
Agar–electrode impedance of dry textile electrodes. FS structure at the pressures of 10 mmHg, 20 mmHg, and 30 mmHg (~ 1.33 kPa, ~ 2.66 kPa, and 3.99 kPa, respectively). The frequency was at 5 Hz

Dry electrodes have shown great potentials for getting high-quality epidermal biopotential signals, including ECG, EMG, and EEG, in various conditions such as dry and wet skin, and during body movement [29]. Since dry textile electrodes do not have an adhesive, a proper pressure must be applied to maintain the skin–electrode contact to improve the quality of recorded biopotential signals [42]. Therefore, we investigated the effect of pressure on skin–electrode impedance. We selected flat textile substrates made of silver yarn since all the conductive coating materials listed in Table 1 could be successfully printed on this type of substrate without cracks. Pressures of 10 mmHg, 20 mmHg, and 30 mmHg (~ 1.33 kPa, ~ 2.66 kPa, and 3.99 kPa, respectively) were chosen, as they are within the optimal pressure range for compression applications of electrodes [34, 35]. We found that the pressure had little effect on the impedance of dry textile electrodes with different conductive coating materials (Fig. 7). Thereafter, the pressure of 20 mmHg (~ 2.66 kPa) was chosen for the following ECG measurement. This pressure has been shown to deliver the best signal fidelity for ECG applications [42].

#### Effect of temperature

In previous studies with gel electrodes, it has been shown that the resistive and capacitive components of the skin–electrode interface increase with decreasing temperature [43]. This observation was made for the temperature ranges 26–36 °C. This change in impedance has been suggested to be correlated to the change in temperature, rather than changes in blood flow [43]. In this study, the temperature was kept at consistent room temperature of 23 °C, and no fluctuations were presented either to the agar sample or the user being tested. Therefore, we did not account for or investigate the effect of temperature, considering the observed inverse linear relationship of skin–electrode impedance to changes in temperature [43].

#### Long-term impedance stability

**Fig. 8:**
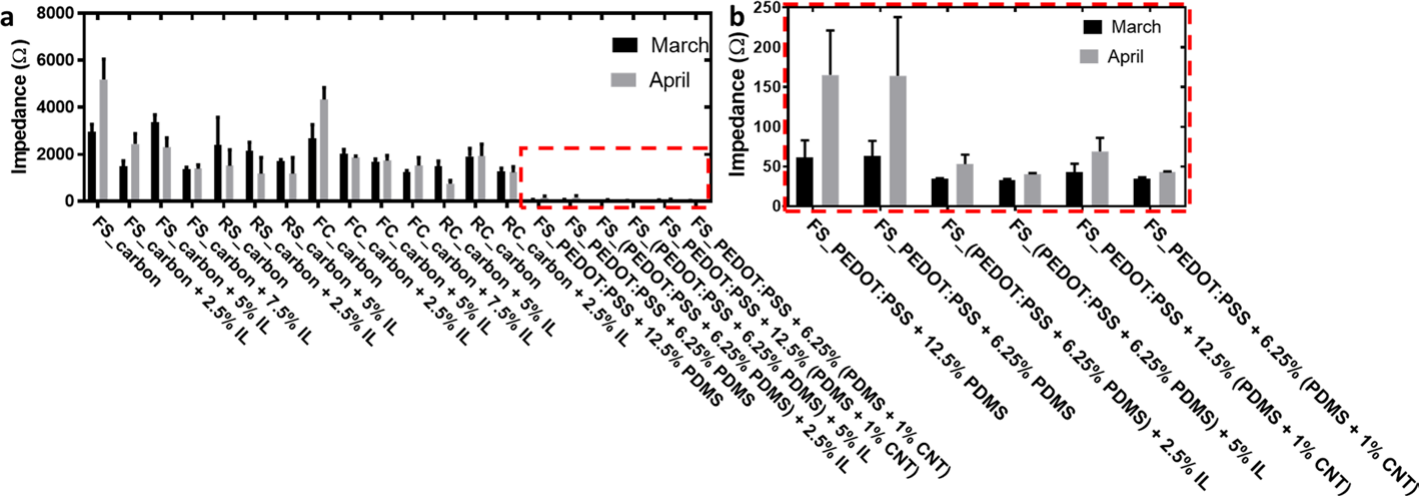
Long-term impedance stability. **a** Dry textile electrodes with various coatings and substrates and **b** zoom-in of PEDOT:PSS-contained electrodes. The pressure was 20 mmHg (~ 2.66 kPa)

The impedance stability performance of the dry electrodes over time is another important concern for the real application of smart garments [36]. We evaluated the deterioration of the dry textile electrodes by quantifying their impedance change within one month. The dry textile electrodes with IL/CNTs-contained coatings maintained their impedance well (Fig. 8). In contrast, dry textile electrodes with pure carbon coatings or PEDOT:PSS + PDMS coatings showed obvious impedance increases.

### Demonstration of ECG quality

**Fig. 9:**
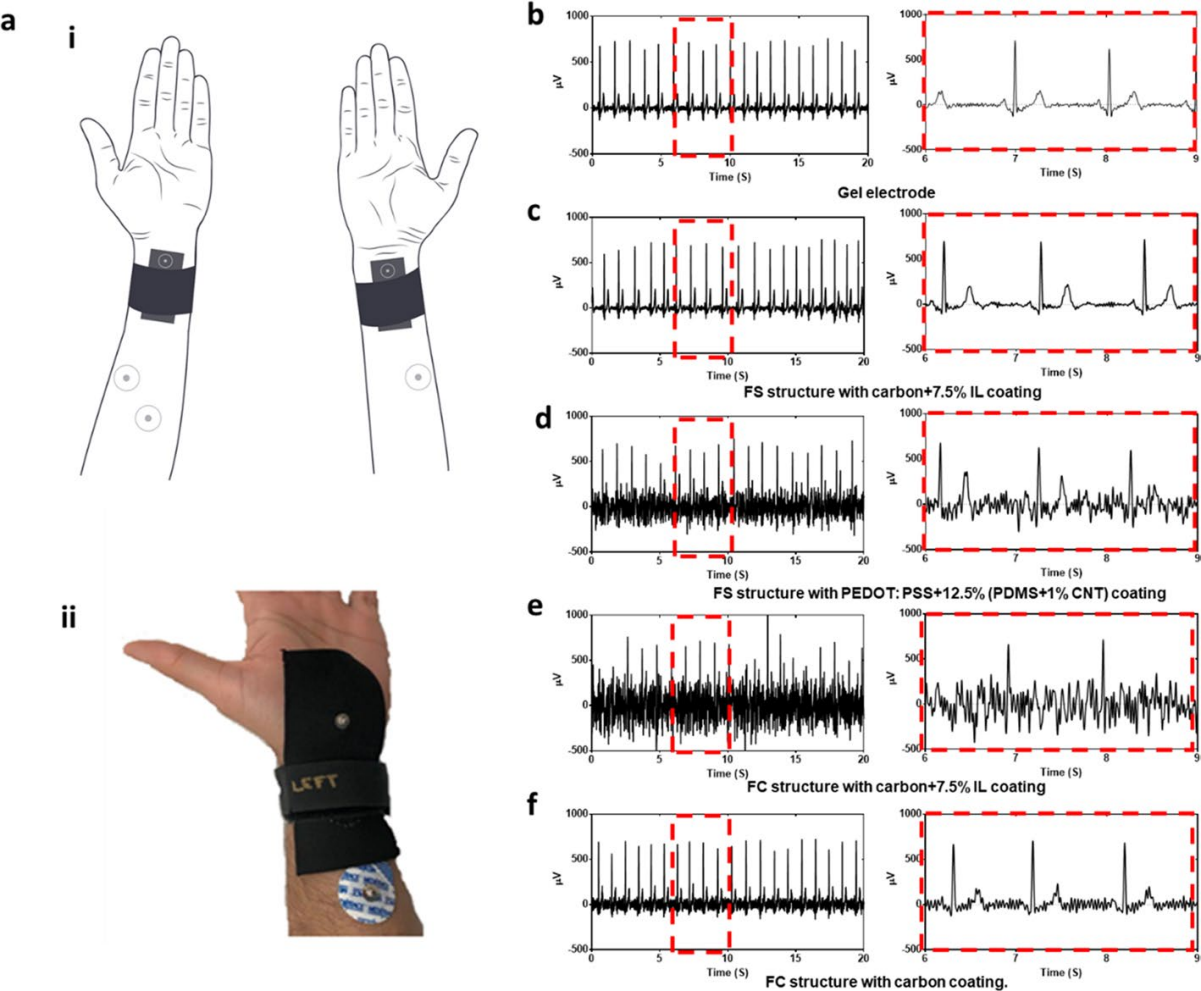
ECG measurement. **a** ECG measurement setup (**i**) and photo of electrodes adhered to the skin (**ii**). Data of ECG monitoring using **b** gel electrode, **c** dry textile electrode in FS structure with carbon + 7.5% IL coating. **d** Dry textile electrode in FS structure with PEDOT: PSS + 12.5% (PDMS + 1% CNT) coating. **e** Dry textile electrode in FC structure with carbon + 7.5% IL coating. **f** Dry textile electrode in FC structure with carbon coating

We evaluated the ECG quality on human skin using the electrodes shown in Fig. 8. Here, we selected dry textile electrodes in FS structure with carbon + 7.5% IL coating, FS structure with PEDOT:PSS + 12.5% (PDMS + 1% CNT) coating, and FC structure with carbon + 7.5% IL coating for ECG monitoring due to their relative good long-term stability (Fig. 8). The dry electrodes were stored dry at room temperature for one month. Five replicates of each electrode were used in order to confirm the presented results. The electrodes were placed on the subject’s arm as shown in Fig. 9a, along with two commercial disposable Ag/AgCl electrodes as the gold standard simultaneously recorded as the second channel, and a third Ag/AgCl electrode as the driven ground electrode. The pressure was applied at 20 mmHg (~ 2.66 kPa) through a stretchable compression band and measured with a PicoPress® sensor. Although the dry textile electrode in FS structure with PEDOT:PSS + 12.5% (PDMS + 1% CNT) coating showed good long-term stability and an extremely low impedance (~ 50 Ω, Fig. 8), it did not present the best ECG signal quality (Fig. 9d). In contrast, the dry textile electrode in FS structure with carbon + 7.5% IL coating showed a relatively high impedance (~ 1500 Ω, Fig. 8), but it presented a similar ECG spectrum (Fig. 8c) as the standard gel electrode (Fig. 9b). Conversely, the dry textile electrode in FC structure with carbon + 7.5% IL coating showed a relatively high impedance (~ 1500 Ω, Fig. 8), but it presented an ECG spectrum (Fig. 8e) with poor signal quality. In addition, we selected dry textile electrodes in FC structure with carbon coating as the representative electrodes without good long-term stability. We noticed that with a high impedance (~ 3000 Ω, Fig. 8) and a poor long-term stability (with a ~ 75% impedance increase in 30 days, Fig. 8), the dry textile electrode in FS structure with carbon coating showed a better ECG spectrum (Fig. 9f) than the FS structure with PEDOT:PSS + 12.5% (PDMS + 1% CNT) coating (Fig. 9b).

**Fig. 10:**
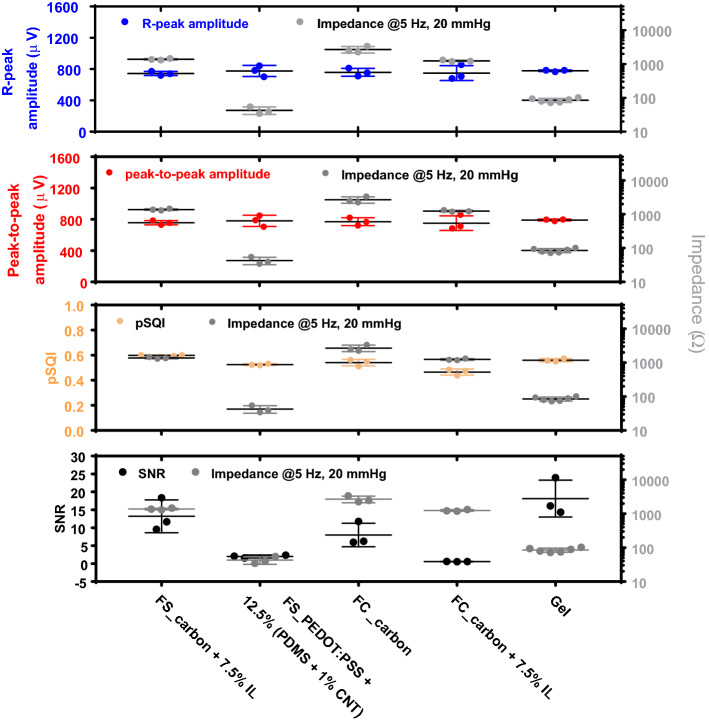
ECG signals quality metrics for dry textile electrodes. Different knitted structures and conductive coating materials

We further evaluated ECG signals based on the signal quality metrics of pSQI values, R-peak amplitudes, peak-to-peak amplitudes, and SNR values (Fig. 10). Although the impedance of dry textile electrodes varies based on the coating conductive materials, their pSQI values, R-peak amplitudes, and peak-to-peak amplitudes are all relatively stable and independent of the contact impedance. The relatively similar pSQI values indicate that the QRS complex was well-captured by all dry textile electrodes. We found that the dry textile electrode in FS structure with PEDOT:PSS + 12.5% (PDMS + 1% CNT) coating has a comparable impedance level to the standard gel electrodes but a significantly lower signal-to-noise ratio (SNR) value. In contrast, the dry textile electrode in FS structure with carbon + 7.5% IL coating showed a significantly higher impedance level than the standard gel electrodes but a comparable SNR value (Fig. 10). Therefore, in dry contact textile electrodes, lower impedance was not correlated to improvements in ECG signal performance, which is contrary to previous findings [7, 28, 29].

## Discussion

In this work, we, for the first time, provided a standard testing procedure and framework for skin–electrode impedance measurement for the development of novel contact textile electrodes. From the experimental results, we found that carbon-contained conductive materials with proper concentration of IL (e.g., ≤ 5%) showed good printability on all dry textile structures (i.e., FS, RS, FC and RC). The PEDOT:PSS + PDMS-contained conductive materials only showed printability on the FS structure. During impedance tests, we found the electrode impedance decreased as the increase of frequency; PEDOT:PSS-contained conductive coatings showed a gel-like electrode impedance, which is much smaller than that of carbon-contained conductive coatings. In addition, for carbon-contained conductive coatings, the electrode materials and structure have little impact on the electrode impedance. Similarly, pressure has no impact on the electrode impedance. As well we found dry textile electrodes with IL/CNTs-contained coatings showed good long-term impedance stability. During ECG measurements, the lower impedance of dry contact textile electrodes with PEDOT:PSS-contained conductive coatings was not correlated to improvements in ECG signal performance.

Based on the results of this study, impedance cannot be used as a single factor to predict the performance of the materials or structures of textile-based dry contact electrodes for electrophysiological applications. The study highlights the importance of considering the electrophysiological application signal, in this case electrocardiography. To that effect, we have demonstrated the relation between electrode–skin impedance and signal quality was investigated using R-peak and peak-to-peak amplitudes as well as pSQI and SNR (Fig. 10). All these parameters are quantifiable attributes of an ECG signal which can provide insight into the performance of the textile-based dry contact electrode.

This work focused on studying the skin–electrode impedance of dry textile electrodes and its potential impact on ECG performance. Other performance of dry textile electrodes such as the motion artifact, electrodes’ breathability and long-term skin irritation of current dry textile electrodes, are worth being explored. Their performance under sweating conditions and stretching while implemented in a textile form factor needs to be investigated for the practical usability considerations. Factors such as textile stretching and wash cycles would definitely impact the performance of dry electrodes. The results and finding of our research would allow us and other researchers to select the best performance dry-electrode formulation and textile substrate for their application and study their life-cycle performance using various wash and wear cycles. In our case, this would be investigated in our future studies depending on the application (ECG, EMG, EOG, and EEG monitoring) of dry electrodes and the textile form factor (sleeve, chest band, underwear, bra, tank top, head band, legging, etc.) since each of these form factors would require various amount of stretchability during wear cycles and different number of wash cycles it was not investigated in depth for this study. Also, it is worth exploring why higher concentrations of IL in the conductive material have a negative effect on the screen printing. In addition, potential biocompatibility and cytotoxicity considerations (mainly originated from the cation) of IL-contained dry textile electrodes need to be investigated over long-term wearing. Currently, there are many efforts pursuing the development of novel formulations of IL with large and bulky cations that can be entrapped in a polymeric matrix with stable and biocompatible features [8]. Before, during and immediately after application of dry electrodes, the skin irritation observation test was performed [44]. No skin irritation was observed for the individual who tested the electrodes for the duration of the testing. Though it is recommended that any materials selected for dry textile electrode long-term usage to be tested and certified based on ISO 10993-5, biological evaluation of medical devices. The current study measured the ECG performance using the dry textile electrode in FS structure with PEDOT:PSS + 12.5% (PDMS + 1% CNT) coating. More ECG tests need to be conducted on dry textile electrodes with gel-electrode-like low impedance to better understand how the low impedance determines the quality of ECG monitoring. In addition, our study focused on the ECG monitoring scenario, other biopotential monitoring (e.g., EMG, EEG, and EOG) using dry textile electrodes are worth being explored. These explorations are expected to provide more useful guidelines to design dry textile electrodes for other biopotential monitoring through a universal/standard evaluation protocol.

## Conclusions

In this paper, we studied and compared the impedance and performance of dry textile electrodes under different factors of substrate and structure, conductive coating materials, pressures, and frequencies. As well we explored their impedance durability for potential long-term applications in wearables. Unlike previous studies that focused on one conductive material across different testing platforms and parameters to evaluate the skin–electrode impedance, this is the first comprehensive study on a wide range of reported materials and their combinations using a skin model. The study has provided a standard testing procedure and framework for the classification of any newly developed materials, substrates or structures for dry contact electrodes, above and beyond dry contact textile electrodes. As well the study has provided a guideline for attributing the performance of dry textile electrodes in practical applications such as ECG monitoring. For example, a raised 3D electrode surface was expected to produce a lower impedance due to their better contact with the skin, while our experimental results did not indicate such a significant tendency. The pressure had little impact on the electrode impedance. In addition, from the impedance tests, we found the electrodes in FS structure with (PEDOT:PSS + 6.25% PDMS) + 2.5% IL coating, (PEDOT:PSS + 6.25% PDMS) + 5% IL coating, PEDOT:PSS + 12.5% (PDMS + 1% CNT) coating, and PEDOT:PSS + 6.25% (PDMS + 1% CNT) coating showed a low impedance level (~ 50 Ω), which is comparable the standard gel electrodes. However, the lower impedance of dry contact textile electrodes [e.g., in FS structure with PEDOT:PSS + 12.5% (PDMS + 1% CNT) coating] cannot guarantee a better ECG performance. In contrast, dry textile electrodes with high impedance (e.g., in FS structure with carbon + 7.5% IL coating, ~ 1500 Ω), presented a high-quality ECG spectrum, similar to that of the standard gel electrode. Therefore, impedance alone should not be considered as the primary indicator for the quality of dry contact electrodes for biopotential recordings.

This study is a culmination of a number of efforts within the field of materials for smart textiles in continuous electrophysiological monitoring, specifically, electrocardiogram. This study is the first of its kind that contributes to the body of scientific knowledge by comparing the variety of materials found in the literature, serving as a comprehensive comparison of these proposed possibilities. These studies have been referenced and used as a logical framework for the selection of materials with the highest promise, towards a meaningful assessment of their efficacy and feasibility. The study not only assessed their electrical performance, but also provided an understanding of how these proposed materials can be produced for continuous monitoring applications, with overviews on their durability after being produced. As well, the study has explored the introduction of new variants through the inclusion of ionic liquids. This collective effort was designed as such to provide a thorough investigation in the field of dry contact textile electrodes, paving the way and encouraging further research in the field, for other electrophysiological applications such as EMG, EEG and EOG. Beyond new applications, there are continuing avenues for this paper, such as, biocompatibility/cytotoxicity considerations based on ISO standards, long-term material durability during wash, wear and tear that an everyday textile would undergo. Though these are beyond the scope of the project, and will be explored in future efforts.

## Methods

The aim of this study was the assessment of the electrical impedance property and performance of dry contact textile electrodes. This was done for a number of materials that have been highly reported and used for on body continuous monitoring applications, both in textiles and printed electronics. In this pursuit a number of the proposed materials that were tested did not successfully translate to textile substrates, which has been documented in the study. For the materials that were successfully implemented, impedance measurements were taken. The literature suggests, the difference between dry contact and conventional adhesive gel electrodes, lies within the skin–electrode impedance profile. In order to systematically approach this challenge, we created a standard testing procedure, which controlled parameters that could affect impedance, namely electrode size, contact pressure, and testing medium (agar). This standard testing procedure was also a helpful and useful outcome of this study, providing researchers a guideline for their future research, development and testing in this area.

### Fabrication of dry textile electrodes

Using a flatbed knitting machine (Stoll, Reutlingen, Germany) silver-plated nylon and carbon-contained nylon conductive yarns (Myant Inc., Canada) were knitted into four different structures; flat textile electrodes made of silver yarn (*FS* Sample code), raised 3D textile electrodes made of silver yarn (*RS* Sample code), flat textile electrodes made of carbon yarn (*FC* Sample code) and raised 3D textile electrodes made of carbon yarn (*RC* Sample code).

These two conductive yarns (namely silver-plated and carbon-contained nylons) are the most common yarn-based conductive materials applicable in textile electrodes. They could also be mass-produced into the yarn form. Anisotropic conductive thermoplastic adhesive (EXP 2650-50, Creative Materials), carbon paste (DuPont™ PE671), carbon black (Cabot Corporation), carbon nanotube (CNT, SilQuan-C, NanoQuan), ionic liquid [1-Ethyl-3-methylimidazolium bis(trifluoromethylsulfonyl) imide, 99%, IL-0023, Iolitec], PDMS (SYLGARD™ 184, Dow Corning Corporation), (Poly(3,4-ethylenedioxythiophene)-poly(styrenesulfonate) (PEDOT:PSS, Orgacon EL-P501, Orgacon), Graphene (N006-P, Angstron Materials) and Neoprene pellets [Neoprene Polychloroprene, DuPont (pellets)] were used as ingredients of the different conductive pastes made for this study. The conductive pastes were either solely carbon-based or contained the polymers PEDOT:PSS, PDMS, and/or ionic liquid, and their detailed compositions are summarized in Table 1 and Additional file 1: Table S2. To make a homogeneous conductive paste, first the ingredients were mixed at 3500 rpm for 30 s using a speed mixer [SpeedMixerTM (DAC 150.1 FV)]. The paste was placed on a custom-made silk screen mesh (156 mesh count with a template being 2.54 cm by 2.54 cm square, large enough to completely cover the circular textile electrode surface) and spread through the screen via a squeegee to form a smooth surface on a glossy-finish heat-transfer paper (Additional file 1: Figure S1). The curing condition of each layer depended on the composition of the conductive dry-electrode paste. The number of printing layers and curing conditions for each layer were per the recommendations of the materials supplier. Material compositions, number of ink layers used, and heat curing settings used in this study are given in Table 1 and Additional file 1: Table S2 with and without successful screen printing, respectively. The selection criteria of successful printing are based on the fact that printed electrodes maintain fidelity and mechanical durability when bent 180° for 50 times. The optimal method for the screen printing of the conductive pastes was on a heat-transfer paper, rather than direct screen printing on a textile substrate. To allow for proper conductive adhesion to the textile substrate, an anisotropic conductive thermoplastic adhesive was printed on top of the cured conductive layers for paste formulations not containing PDMS. After printing and curing the required number of layers for each coating paste on a heat-transfer paper, the prints were heat-transferred (250 °F, for 30 s, at 90 psi) from the paper to the textile, such that the adhesive was in contact with the conductive textile portion. Since PDMS has poor adhesive properties, pastes containing PDMS were printed on top of the cured layer of a thermoplastic adhesive and then manually peeled from the paper and applied to the textile substrate. The printed electrodes were then laminated onto the textile substrates, with the thermoplastic adhesive side of the electrode coming in contact with the textile substrate, and then adhered via heat-transfer. To complete the electrodes, a non-conductive adhesive film (BEMIS Company, Inc.) was applied as the last step via heat-transfer. This was designed as such to ensure an equal functional (conductive component) contact area for all electrodes, equivalent to a circle with a diameter of 2 cm. As well, for all heat-transfers, the settings of temperature, time and pressure were kept the same to ensure consistency in process. This helped prevent non-homogenous factors that could impact the material characteristics, ultimately affecting performance and durability of the electrodes. This Fig. 2, h shows flat and raised 3D textile electrodes with screen printing of the conductive paste. All developed electrodes were then placed in a standard atmosphere for 24 h [relative humidity (RH) = 65 ± 2% and *T* = 20 ± 2 °C] before their electrical properties were measured. Digital microscope (Oitez USB microscope) was used to evaluate the morphological characteristics of textile electrodes.

### Skin–electrode impedance measurement

The skin–electrode impedance of the dry textile electrodes produced with various coatings was recorded using an Ivium Potentiostat (Ivium Technologies, Netherlands) in the frequency ranges from 1 Hz to 10 kHz. For consistency in the testing of electrodes, which can be significantly affected by intra- and inter-subject skin impedance variations, a human skin model optimized to match test results on human skin was created [33]. Briefly, this was achieved by mixing a solution of 4.5% agar, 0.97% NaCl, and deionized water on a hot plate until boiling, after which it was poured into a glass container and cooled until the mixture solidified. A new batch of agar was prepared using the mentioned recipe for each daily experimental and each agar batch was used for no longer than 7 h. This was done to prevent drying of the agar simulated skin model, which would impact impedance recordings. Three dry textile electrodes were placed 7 cm apart on the agar skin model using the configuration reported in studies assessing skin–electrode contact impedance [12] (Additional file 1: Figure S2). With the 2-electrode test (Additional file 1: Figure S2b), the Counter (C) and Reference (R) cables are connected to Electrode 1. The Sense (S) and Working (W) cables are connected to Electrode 2. For the 3-electrode test (Additional file 1: Figure S2a), the configuration is the same except the Working cable is connected to Electrode 3. For both, the Ivium supplies constant amplitude current through the W and C cables, while simultaneously reading voltage through the S and R cables. The 2-electrode test obtains the impedance of the skin tissue (including subcutaneous tissue), which in this case would be agar, as well as Electrode 1 and Electrode 2. The 3-electrode test obtains the impedance of the skin tissue (including subcutaneous tissue), which in this case would be agar, and Electrode 1. Therefore, the impedance of Electrode 2 alone can be calculated by subtracting the impedance obtained by the 3-electrode test from the 2-electrode test.

The electrodes were given 5 min to settle on the agar before recording impedance characteristics. The electrode was considered settled when the impedance values ceased to continually increase or decrease with each measurement. A pressure (e.g., 20 mmHg or ~ 2.66 kPa) was applied on the electrodes, using a weight with the area of the weight in contact with the electrodes equal to that of the electrode face. This pressure is typically used in textile biopotential measurement to provide a secure and comfortable fit with good signal quality [42]. The electrode impedance of each category was measured at least on 3 samples, along with that of hydrogel electrodes measured as the control group.

### Biopotential electrocardiogram (ECG) measurements

The electrodes were compared with gold standard hydrogel electrodes (Kendall, Covidien) for ECG measurement. ECG of 40 s duration was collected for each electrode to demonstrate the effect of coating on electrode performance for signal fidelity and illustrate the performance of the textile electrodes in comparison to hydrogel electrodes. ECG measurements were performed on one individual to minimize inter-subject impedance variability due to differences in skin impedance.

A low-power, 24-bit analog front-end ECG acquisition board (ADS1293, Texas Instruments) was used to collect ECG signals at 853 Hz. The acquisition board has an instrumentation amplifier (3.5 × gain), followed by a sigma-delta modulator, and digital filter. The chip contains three programmable fifth-order Sinc filters with decimation rates of 4, 5, and 6, respectively. All signals were filtered with two fourth-order Butterworth filters, first using a high-pass filter (*f*_c_ = 0.5 Hz), then using a low-pass filter (*f*_c_ = 50 Hz). The Pan–Tompkins algorithm was used to find R-peaks [45]. MATLAB R2016b was used for all post-processing. The following metrics for signal quality were extracted from the filtered ECG:

1. Amplitude: the distance, in µV, from the baseline signal to each R-peak. The baseline refers to the mean value of the entire signal.
2. Peak-to-peak amplitude: the distance, in µV, between consecutive from the S- to the R-peak [46].
3. pSQI: the relative power of the QRS complex, $\frac{{\mathop \smallint \nolimits_{{5\;{\text{Hz}}}}^{{15\;{\text{Hz}}}} P\left( f \right){\text{df}}}}{{\mathop \smallint \nolimits_{{5\;{\text{Hz}}}}^{{40\;{\text{Hz}}}} P\left( f \right){\text{df}}}}$ [47].
4. BasSQI: the relative power of the baseline signal, 1 − $\frac{{\mathop \smallint \nolimits_{{0\;{\text{Hz}}}}^{{1\;{\text{Hz}}}} P\left( f \right){\text{df}}}}{{\mathop \smallint \nolimits_{{0\;{\text{Hz}}}}^{{40\;{\text{Hz}}}} P\left( f \right){\text{df}}}}$ [47].

To ensure that the use of textile electrodes did not alter the shape of the frequency distribution of the measured ECG, Welch’s estimated power spectral densities (PSDs) were compared using Pearson’s correlation coefficient *R*^2^ for each of the ECG samples. This would help mitigate the influence of confounding factors that can affect the signal quality or skin impedance between measurements, such as abrasion of the stratum corneum during removal and replacement of electrodes, motion, skin hydration, and sweating [48–52].

## Supplementary Information

**Additional file 1: Figure S1.** Electrode screen printing. (a) The screen-printing setup with the screen and template pattern, and red squeegee. (b) The resulting deposition of conductive paste on heat transfer paper. **Figure S2.** Ivium connection setup for impedance testing. (a) 3-electrode configuration (b) 2-electrode configuration. **Figure S3.** The agar–electrode impedance. (a) RS structure and (b) RC structure with various conductive coating materials in the frequency range of 1–10000 Hz. The pressure was 20 mmHg (~ 2.66 kPa). **Table S1.** Summary of current solutions to dry textile electrodes for biopotential monitoring. **Table S2.** Coating formulations unsuccessfully screen-printed onto dry textile electrodes.

## Data Availability

The datasets during and/or analysed during the current study are available from the corresponding author on reasonable request.

## Abbreviations

Ag/AgCl: Silver/silver chloride
ECG: Electrocardiography
CVD: Cardiovascular disease
ICP: Intrinsically conductive polymers
PEDOTPSS: Poly(3,4-ethylenedioxythiophene)-poly(styrenesulfonate)
IL: Ionic liquid
PDMS: Polydimethylsiloxane
CNT: Carbon nanotube
FS: Flat textile electrodes made of silver yarn
RS: Raised 3D textile electrodes made of silver yarn
FC: Flat textile electrodes made of carbon yarn
RC: Raised 3D textile electrodes made of carbon yarn
EEG: Electroencephalography
EMG: Electromyography
SNR: Signal-to-noise ratio
RH: Relative humidity
NaCl: Sodium chloride
pSQI: The relative power of the QRS complex
BasSQI: The relative power of the baseline signal
PSD: Power spectral density
