# Supplementary material for: Evaluation of dry textile electrodes for long-term electrocardiographic monitoring
Source: Biomed Eng Online. 2021 Jul 12;20:68. doi: 10.1186/s12938-021-00905-4 (PMC8274056; doi:10.1186/s12938-021-00905-4)
Supplement: Supplementary file 1 — Additional file 1: Figure S1. Electrode screen printing. (a) The screen-printing setup with the screen and template pattern, and red squeegee. (b) The resulting deposition of conductive paste on heat transfer paper. Figure S2. Ivium connection setup for impedance testing. (a) 3-electrode configuration (b) 2-electrode configuration. Figure S3. The agar–electrode impedance. (a) RS structure and (b) RC structure with various conductive coating materials in the frequency range of 1–10000 Hz. The pressure was 20 mmHg (~ 2.66 kPa). Table S1. Summary of current solutions to dry textile electrodes for biopotential monitoring. Table S2. Coating formulations unsuccessfully screen-printed onto dry textile electrodes. [file 12938_2021_905_MOESM1_ESM.docx]

# Additional file 1


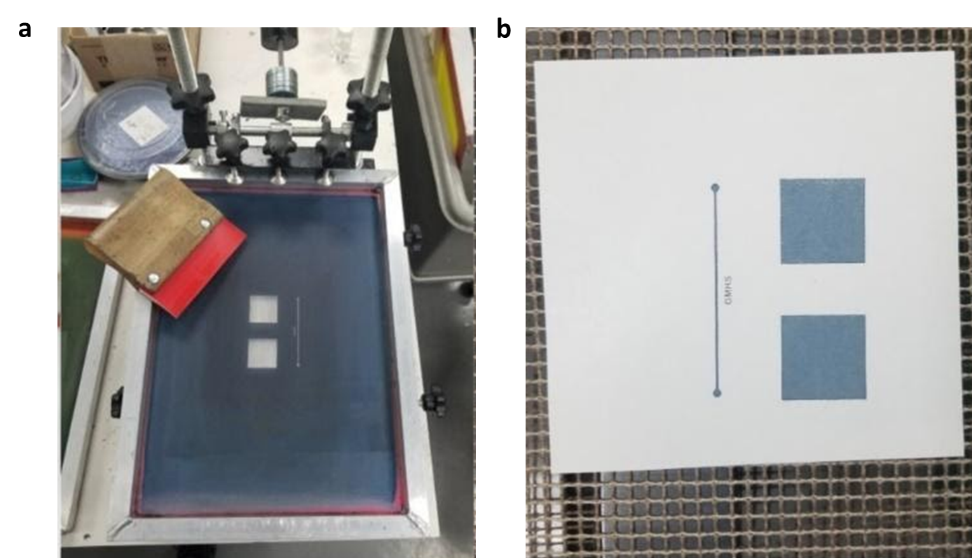


Figure S1. Electrode screen printing. (a) The screen-printing setup with the screen and template pattern, and red squeegee. (b) The resulting deposition of conductive paste on heat transfer paper.


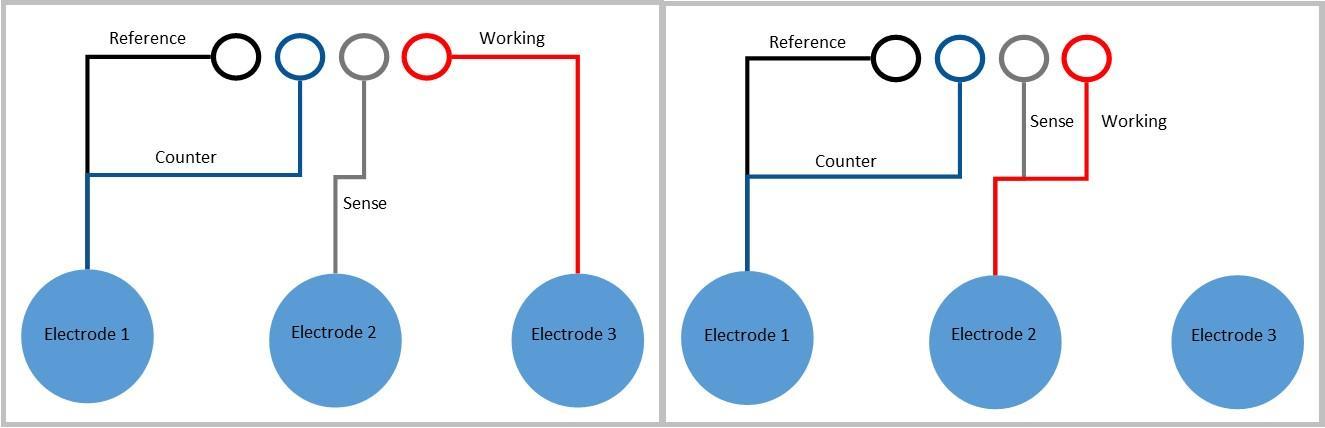


Figure S2. Ivium connection setup for impedance testing. (a) 3-electrode configuration (b) 2-electrode configuration.


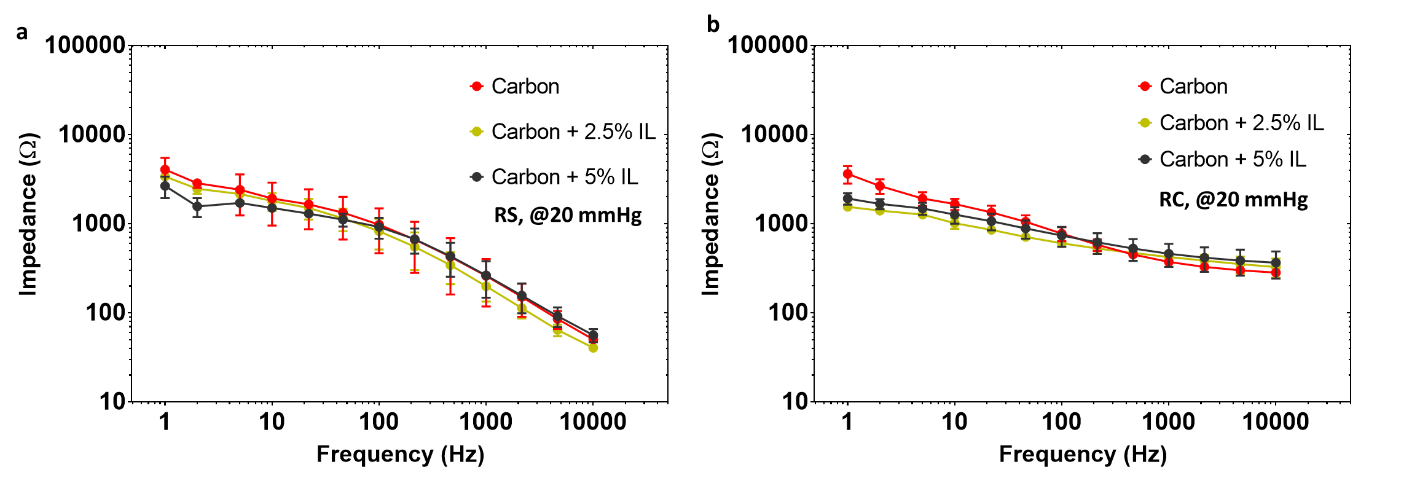


Figure S3. The agar-electrode impedance. (a) RS structure and (b) RC structure with various conductive coating materials in the frequency range of 1-10000 Hz. The pressure was 20 mmHg (~2.66 KPa).

Table S1. Summary of current solutions to dry textile electrodes for biopotential monitoring.

| **Conductive Coating Material** | **Textile Substrate** | **Impedance Test Parameters (Frequency, Pressure)** | **Electrical Properties** | **Application and Ref.** |
| --- | --- | --- | --- | --- |
| Screen-printed silver paste | Nonstretch fabric | Impedance measured at 1-200 Hz frequency, <20mmHg (~2.66 KPa) pressure | Impedance: 10 - 800 kΩ.  Sheet resistance: 0.01 - 60 mΩ/sq | ECG([16](#_ENREF_16)) |
| Screen-printed silver/silver-Chloride ink | Vista-maxx propylene-based elastomeric meltblown nonwoven fabric | 100 Hz, <30 mmHg (~3.99 KPa) pressure | Impedance: 150 kΩ* | ECG[^7^](#_ENREF_7) |
| Screen-printed silver paste | Escalade: 46/16/38 Cotton/Polyester/  Lycra® | N/A | Resistance: < 1 kΩ | ECG, EMG([53](#_ENREF_53)) |
| Screen-printed silver paste | Polyester/Cotton (65/35, 2X1 twill) with polyurethane interface layer | N/A | Sheet resistance: 49.4 mΩ/sq | FES([20](#_ENREF_20)) |
| Film-casted silver nanowires | PDMS film | 500 Hz, 14 mmHg (~1.86 KPa) (selected from range) | Impedance: 700 kΩ* | ECG, EMG([54](#_ENREF_54)) |
| Film-casted silver nanowires | PDMS film | N/A | Sheet resistance: 0.24 Ω/sq | Strain sensor([55](#_ENREF_55)) |
| Dip-coated graphene+PEDOT:PSS | Cotton | N/A | Sheet resistance: 3.67 kΩ/sq (1 layer), 11.51 Ω/sq (7 layers) | ECG([56](#_ENREF_56)) |
| Chemical vapor deposition growth of graphene | Copper | 20 Hz (selected from range), Pressure from adhesive | Impedance: 65.82 kΩ | ECG([57](#_ENREF_57)) |
| Dip-coated graphene oxide | Nylon® | 10 Hz, 1kHz, Pressure from adhesive | Impedance: 87.6 kΩ (10 Hz), 11.6 kΩ (1 kHz) | ECG([58](#_ENREF_58)) |
| Dip-coated graphene oxide | Nylon® | N/A | Resistance: 200 kΩ* (1 coat), 2 kΩ*(6 coats) | ECG([59](#_ENREF_59)) |
| Compression-molded EPDM rubber with carbon, stainless steel fibers, carbon nanotubes | EPDM matrix | 10 Hz, Pressure from adhesive | Impedance: 1–10 MΩ·cm^2^ | ECG, EEG ([60](#_ENREF_60)) |
| Roller-painted carbon black | Woven cotton with and without interlaced stainless steel yarns | N/A | Sheet resistance: 1.25 kΩ/sq (cotton, 15.96 wt% CB), 0.016 kΩ/sq (cotton/stainless steel, 16.88 wt% CB) | Smart textiles([18](#_ENREF_18)) |
| Molded carbon black | PDMS matrix | 33 Hz, “medium pressure”, 2cm diameter electrodes | Impedance: 274 - 378 kΩ | ECG, EMG([19](#_ENREF_19)) |
| Molded MWCNTs | PDMS matrix | 30 Hz, Pressure from adhesive | Impedance: 348 kΩ (micropillar structure), 1.52 MΩ (flat structure) | ECG([61](#_ENREF_61)) |
| Molded MWCNTs+Polydimethylsiloxane | PDMS matrix | 1 Hz, Pressure from compression bandage | Impedance: 6 kΩ* | ECG([62](#_ENREF_62)) |
| Brush-painted MWCNTs in tapioca starch paste | Scoured cotton | N/A | Resistance: 380 Ω* (1 layer), 40 Ω* (5 layer) | ECG([63](#_ENREF_63)) |
| Screen-printed PEDOT:PSS+Dimethylsulfoxide+Triton^TM^ x-100 | Nonwoven PET fabric | 10 Hz, Pressure from chest strap | Impedance: 270 kΩ.  Sheet resistance: 5.6 Ω/sq. | ECG([17](#_ENREF_17)) |
| Screen-printed PEDOT:PSS | Mediatex TT ACQ | 10 Hz, Pressure from chest strap | Impedance: 25-32 kΩ (inner ring), 27-32 kΩ (outer ring)  Sheet resistance: 268 Ω/sq | ECG([64](#_ENREF_64)) |
| Dip-coated PEDOT:PSS+Glycerol | Woven cotton and woven polyester (non-stretch) | 20 Hz, 100 Hz (selected from range), Pressure from adhesive | Impedance 40kΩ* (20 Hz), 10 kΩ* (100 Hz) | ECG([65](#_ENREF_65)) |
| Inkjet-printed PEDOT:PSS+EG+GOPS | Coated paper | 1 Hz, Pressure N/A | Impedance 1.40 MΩ, 1.26 MΩ, 1.17 MΩ (1-3 layers) | ECG([66](#_ENREF_66)) |

Table S2. Coating formulations unsuccessfully screen-printed onto dry textile electrodes.

| **Coating Material Composition** | **Layers** | **Heat curing** | **Pass/Fail** |
| --- | --- | --- | --- |
| Graphene | 5 | 140°C, 2 mins | Fail on FS, RS, FC, and RC* |
| Graphene+5% IL | 5 | 140°C, 2 mins | Fail on FS, RS, FC, and RC |
| Neoprene+7% Carbon Black | 4 | 140°C, 6 mins | Fail on FS, RS, FC, and RC |
| (Neoprene+7% Carbon Black)+5% IL | 4 | 140°C, 6 mins | Fail on FS, RS, FC, and RC |
| (Neoprene+7% Carbon Black)+10% IL | 4 | 140°C, 6 mins | Fail on FS, RS, FC, and RC |
| PEDOT:PSS | 2 | 150°C, 10 mins | Fail on FS, RS, FC, and RC |
| PEDOT:PSS+5% IL | 2 | 150°C, 10 mins | Fail on FS, RS, FC, and RC |
| PDMS+1% CNT | 2 | 150°C, 10 mins | Fail on FS, RS, FC, and RC |
| (PDMS+1% CNT) + 10% IL | 2 | 150°C, 10 mins | Fail on FS, RS, FC, and RC |
| (PEDOT:PSS+6.25% PDMS)+7.5% IL | 2 | 150°C, 10 mins | Fail on FS, RS, FC, and RC |

* FS: flat textile electrodes made of silver yarn; RS: raised 3D textile electrodes made of silver yarn; FC: flat textile electrodes made of carbon yarn; RC: raised 3D textile electrodes made of carbon yarn.
